# A naturally occurring feline leukaemia virus subgroup A variant exhibits broad mammalian cellular tropism and utilizes canine thiamine transporter 1

**DOI:** 10.1099/jgv.0.002316

**Published:** 2026-09-29

**Authors:** Yuka Kanemura, So Nakagawa, Takayuki Miyazawa

**Affiliations:** 1 Laboratory of Virus-Host Coevolution, Institute for Frontier Life and Medical Sciences, Kyoto University, 53 Shogoin-Kawaharacho, Sakyo-ku, Kyoto 606-8507, Japan; 2 Department of Molecular Life Science, Tokai University School of Medicine, 143 Shimokasuya, Isehara, Kanagawa 259-1193, Japan; 3 Kyoto Animal-Human-Organism Research Institute (Kyoto AHOI), c/o QUESTION, 390-2 Shimomaruyacho, Nakagyo-ku, Kyoto 604-8006, Japan

**Keywords:** cellular tropism, feline leukaemia virus, feline leukaemia virus subgroup A (FeLV-A), gammaretrovirus, receptor usage, thiamine transporter 1, viral envelope

## Abstract

Feline leukaemia virus subgroup A (FeLV-A) has generally been regarded as having a narrow, predominantly feline host range. Here, we characterize a naturally occurring FeLV-A isolate, strain F8701, that exhibits unexpectedly broad mammalian cell tropism. Using an infectious molecular clone of F8701, we show that F8701 infects canine cells efficiently and can utilize the canine orthologue of thiamine transporter 1, the canonical FeLV-A receptor. Compared with reference FeLV-A strains, F8701 displayed markedly higher infectivity in multiple non-feline cell lines, including canine and human cells. Sequence analysis of the envelope glycoprotein revealed that F8701 carries the previously described 83D/91D di-aspartic acid residue combination in variable region A, together with an additional K105N substitution whose functional contribution remains to be determined. Interference assays confirmed that F8701 belongs to FeLV subgroup A, indicating that broad cellular tropism can occur within a naturally occurring FeLV-A background. Although replication in cultured canine cells was observed, whether F8701 can establish productive infection or transmission in dogs *in vivo* remains unknown. These findings highlight the natural plasticity of the FeLV-A envelope and suggest that receptor compatibility can contribute to the expansion of mammalian cellular tropism, while additional host-level barriers are likely to determine cross-species infection *in vivo*.

## Data availability

The Env nt sequence of the molecular clone derived from FeLV strain F8701 has been deposited in DDBJ under accession number LC939534. The DDBJ entry uses the entry identifier F87A1_env; for clarity and consistency, the molecular clone is designated pF8701 in the present manuscript.

## Introduction

Feline leukaemia virus (FeLV) is a gammaretrovirus associated with a wide spectrum of diseases in domestic cats, including lymphoma, aplastic anaemia and immunodeficiency [1–3]. Based on receptor usage and viral interference patterns, FeLV isolates are classified into several subgroups. Among them, FeLV subgroup A (FeLV-A) is generally considered the principal horizontally transmissible subgroup in nature and serves as a major precursor for other subgroups generated through mutation or recombination [4–11]. Although FeLV-B has also been reported in naturally infected cats and may be transmitted in limited circumstances, usually in association with FeLV-A, FeLV-A remains central to understanding FeLV transmission, subgroup evolution and host range determinants.

For many years, FeLV-A was regarded as having a narrow, predominantly feline host range. Pseudotype-based entry assays showed that most reference FeLV-A strains, including Glasgow-1 and 61E, exhibit limited infectivity in non-feline cells and inefficient infection of canine cells [12]. Thiamine transporter 1 (THTR1), encoded by SLC19A2, was later identified as the primary cellular receptor for FeLV-A, providing a molecular basis for subgroup A entry [13]. However, the cellular host range of FeLV-A cannot be explained simply by the presence of a receptor orthologue, because the efficiency of Env–receptor interactions differs among viral strains and host species.

Previous studies have shown that subtle amino acid substitutions within the FeLV Env glycoprotein, particularly in variable region A (VRA), can markedly influence receptor usage and host range [9, 10, 14]. In particular, a di-aspartic acid residue combination at positions 83 and 91 in VRA has been associated with enhanced utilization of mammalian THTR1 orthologues and expanded infectivity in non-feline cells [14]. Nevertheless, naturally occurring FeLV-A variants that exhibit robust and broad mammalian cell tropism remain poorly characterized, and the extent to which natural Env variation can expand cellular tropism within subgroup A remains incompletely understood.

In this study, we characterize a naturally occurring FeLV-A isolate, strain F8701, that displays unexpectedly broad mammalian cell tropism. Using an infectious molecular clone of F8701, we show that F8701 infects canine cells efficiently and can utilize canine THTR1 as an entry receptor. We further demonstrate that F8701 belongs to interference subgroup A, despite its expanded cellular tropism. These findings provide new insights into the natural plasticity of the FeLV-A envelope and show that broad mammalian cellular tropism can occur within a naturally occurring FeLV-A background.

## Methods

### Cell cultures

All cell lines used in this study were maintained in Dulbecco’s modified Eagle’s medium (DMEM; Sigma-Aldrich, St. Louis, MO, USA) supplemented with 10% heat-inactivated fetal calf serum, penicillin (100 units/ml) and streptomycin (100 µg ml^−1^) (Nacalai Tesque, Kyoto, Japan). Cells were cultured at 37 °C in a humidified atmosphere containing 5% CO_2_. The feline cell lines used were the feline embryonic cell line FEA [15, 16], CRFK cells, AH927 cells and the feline fibroblast S+L− cell line QN10S [12, 17]. Canine cell lines included Madin–Darby canine kidney (MDCK) cells, canine osteosarcoma D17 cells and Cf2Th cells, which were used as a source of canine THTR1 cDNA. To evaluate cellular host range, a panel of mammalian and avian cell lines was employed: human TE671 and 293T cells, bovine MDBK cells, ovine OKBJ cells, equine FHK-T cl3.1 cells, porcine PK15 cells, rabbit SIRC cells, rat C6 and HSN cells, murine MDTF and NIH3T3 cells, mink Mv1-Lu cells and avian DF-1 cells. FHK-T cl3.1 cells [18] were kindly provided by Dr Ken Maeda (National Institute of Infectious Diseases, Japan). For marker rescue assays, FEA(*lacZ*) and D17(*lacZ*) cells were established by transducing the parental cells with an *nlsLacZ* gene vector, as described previously [19].

### Quantification of FeLV long terminal repeat DNA and *env* PCR

To quantify FeLV DNA in infected MDCK cells, real-time PCR was performed using primers targeting the U3 region of the FeLV long terminal repeat (LTR), following the established protocol of Tandon *et al*. [20]. Because these primers amplify the LTR region, the results are described as FeLV LTR DNA copy numbers rather than as proviral DNA copy numbers. Canine GAPDH gene-specific primers were used as an internal control for normalization [21]. Genomic DNA was extracted using a DNeasy Blood and Tissue kit (QIAGEN, Valencia, CA, USA). Standard curves for quantification were generated using serially diluted pFGA-5 plasmid DNA [11]. Real-time PCR was conducted using a Bio-Rad CFX Connect Real-Time System (Bio-Rad, Hercules, CA, USA) with Power SYBR Green PCR Master Mix (Applied Biosystems, Waltham, MA, USA).

For endpoint PCR analysis shown in Fig. 1(b), an *env*-specific region was amplified from genomic DNA extracted from infected MDCK cells. The expected PCR product size was ~2 kbp. PCR products were analysed by agarose gel electrophoresis. All primers used in this study are listed in Table S1 (available in the online Supplementary Material).

**Fig. 1.**
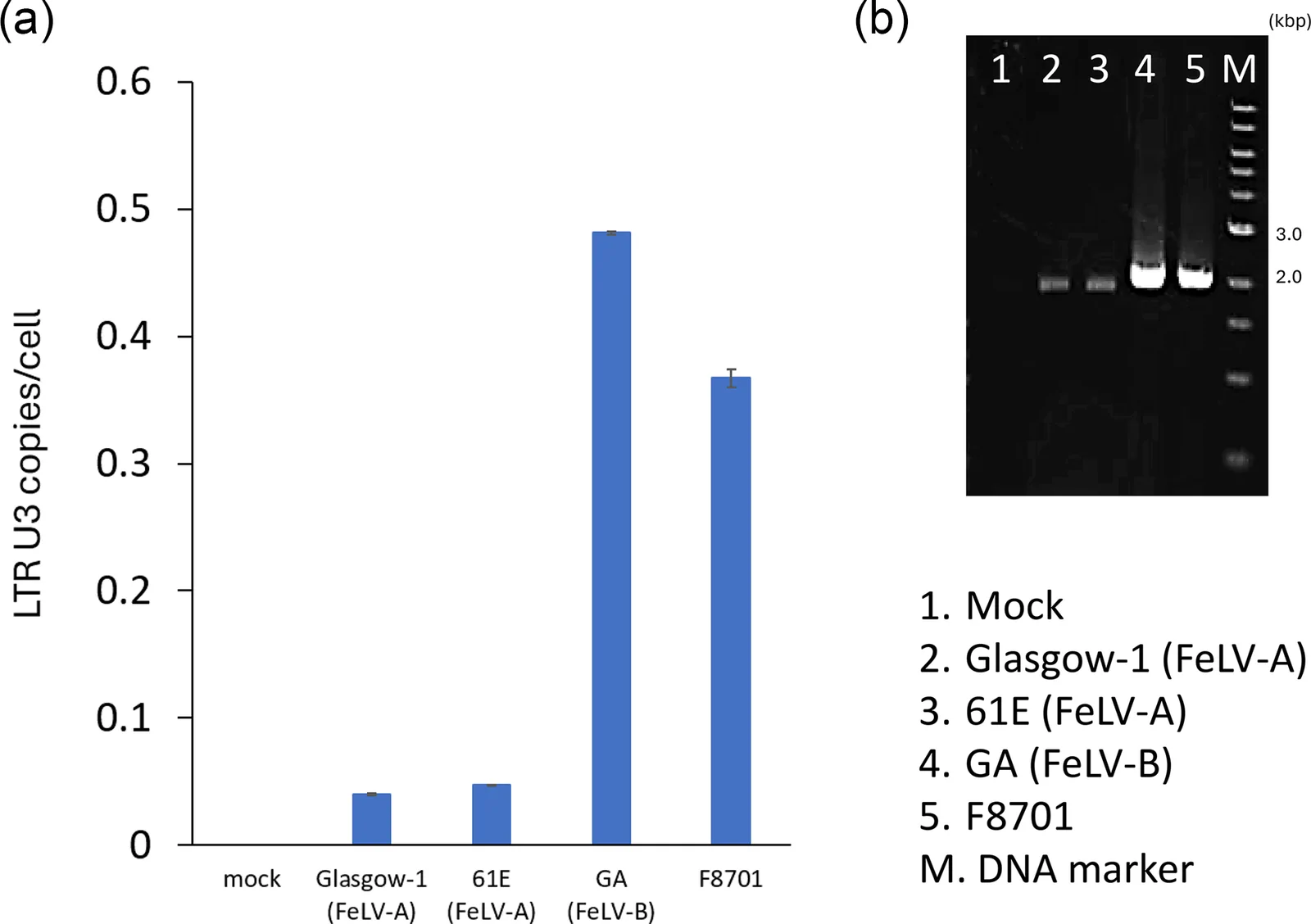
Detection and quantification of FeLV DNA in canine MDCK cells after infection with the parental F8701 isolate. Canine MDCK cells were inoculated with FeLV-A strains Glasgow-1 and 61E, FeLV-B strain GA or virus-containing supernatant from FEA cells infected with the parental FeLV strain F8701. Virus inocula were adjusted to 1.0×10^3^ focus-forming units/well. (**a**) Quantification of FeLV LTR DNA copy numbers by real-time PCR targeting the U3 region at 9 days post-inoculation. Because the primers amplify the LTR region, the results are expressed as FeLV LTR DNA copy numbers. Data represent the mean±sd of technical quantitative PCR (qPCR) replicates (*n*=3) from a single infection experiment. (**b**) Agarose gel electrophoresis of env-specific PCR products amplified from DNA extracted from infected MDCK cells at 9 days post-inoculation. The expected product size was ~2 kbp. Lane M, 1-kbp DNA ladder; lane 1, uninfected control; lane 2, Glasgow-1; lane 3, 61E; lane 4, GA; lane 5, F8701.

### Construction of an infectious molecular clone of FeLV strain F8701

The parental FeLV strain F8701 used in this study was originally described by Nakamura *et al*. [22]. Genomic DNA was isolated from FEA cells infected with FeLV strain F8701. Two overlapping fragments covering the entire FeLV genome were amplified by PCR using PrimeSTAR GXL DNA polymerase (TaKaRa, Shiga, Japan) and primer sets listed in Table S1. The amplified fragments were ligated at the internal BamHI site present in the overlapping region, corresponding to the C-terminal region of pol and the N-terminal region of env, and cloned into the pSP73 vector (Promega, Fitchburg, WI, USA) to generate the infectious molecular clone pF8701 (Fig. 2a). To avoid unnecessary nomenclatural complexity, the infectious molecular clone derived from strain F8701 is designated pF8701, and the virus rescued from this clone is referred to as F8701 throughout this manuscript unless otherwise specified.

**Fig. 2.**
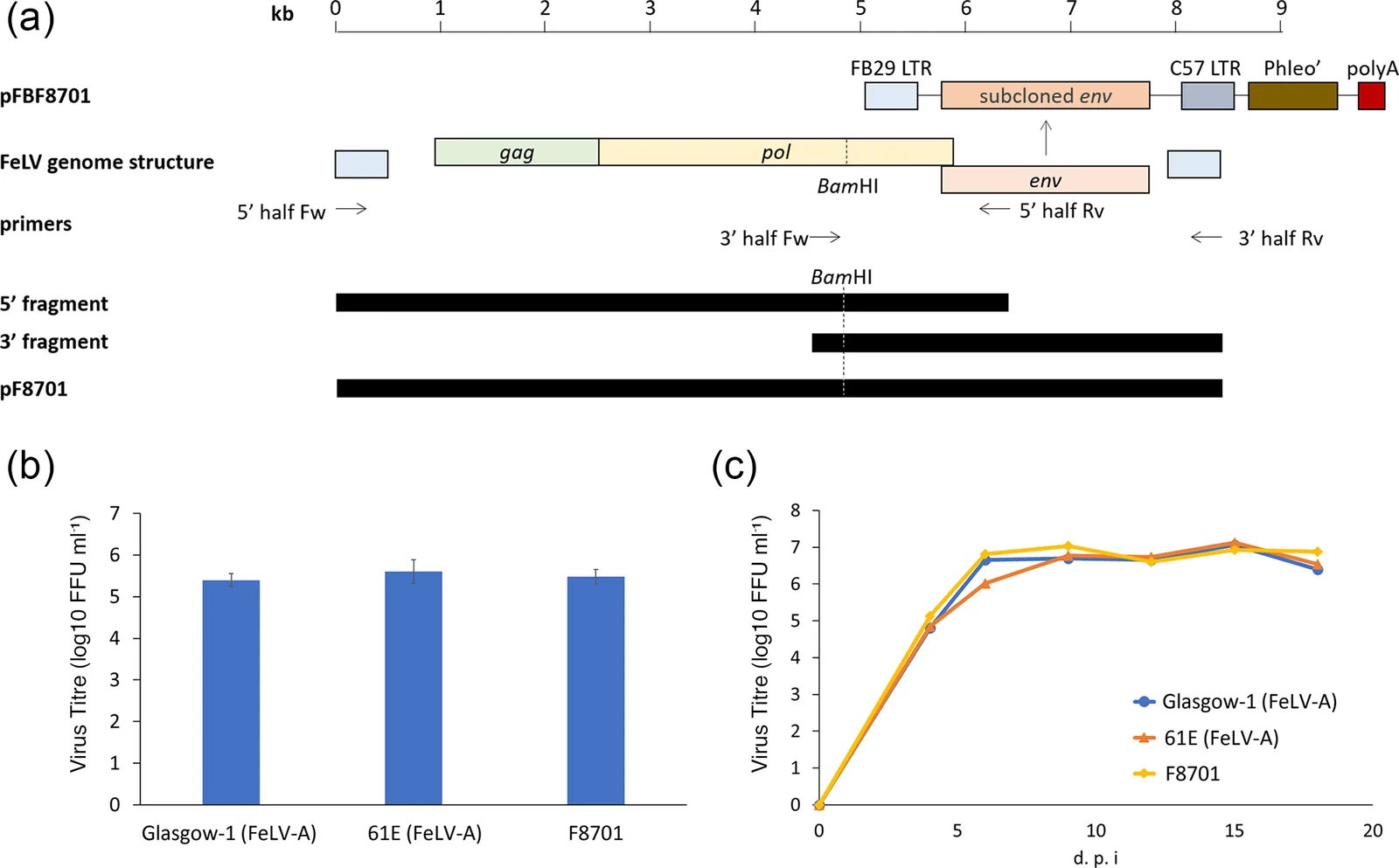
Construction of an infectious molecular clone of FeLV strain F8701 and growth kinetics in feline cells. (**a**) Schematic representation of the construction of pF8701. Two overlapping genomic fragments covering the 5′ and 3′ regions of the F8701 genome were amplified and ligated at the internal BamHI site to generate the infectious molecular clone pF8701. The *env* gene of pF8701 was also subcloned into the pFBFeLV-A backbone to generate pFBF8701 for pseudotype assays. (**b**) Virus production from infectious molecular clones. FEA cells were transfected with molecular clones of Glasgow-1, 61E or F8701, and virus titres in culture supernatants were determined at 14 days post-transfection by the S+L− focus assay using QN10S cells. Data represent the mean±sd of technical replicate wells (*n*=4) from a representative S+L− assay. (**c**) Growth kinetics of FeLV-A strains in FEA(*lacZ*) cells determined by the LacZ marker rescue assay. FEA(*lacZ*) cells were inoculated with Glasgow-1, 61E or F8701 at 1.0×10^3^ focus-forming units (FFU)/well, corresponding to an m.o.i. of 0.001. Culture supernatants were collected at the indicated time points and inoculated onto naïve FEA cells for titre determination. Data are from a single growth-kinetics experiment; each titre was determined using technical replicate wells (*n*=4).

### Plasmids

To construct an expression plasmid encoding F8701 Env, the env gene of pF8701 was amplified by PCR using KOD plus-neo DNA polymerase (Toyobo, Osaka, Japan), and the primers are listed in Table S1. The amplified fragment was digested with XbaI and ClaI and cloned into pFBFeLV-A(FGA-5) to generate pFBF8701, as described previously [12] (Fig. 2a).

For construction of THTR1 expression plasmids, the coding regions of feline and canine THTR1, encoded by solute carrier family 19 member 2, were amplified by reverse transcription PCR (RT-PCR) from FEA and Cf2Th cells, respectively, using primers targeting each open reading frame (Table S1). Each THTR1 fragment was inserted into pEF-MCS-2AGFP, which is derived from pEF-BOS, using NEBuilder HiFi DNA Assembly (New England Biolabs, Ipswich, MA, USA) [23, 24]. The resulting constructs were designated pEF-fTHTR1-2AGFP and pEF-cTHTR1-2AGFP.

### Virus preparation and infectivity assays

Infectious FeLVs, including Glasgow-1, 61E, F8701 and GA, were harvested from the culture supernatants of FEA cells transfected with their respective infectious molecular clones. Glasgow-1, 61E and GA were derived from pFGA-5, p61E and pGAHF, respectively. Virus-containing supernatants were passed through 0.45-µm filters and stored at −80 °C until use.

*lacZ*-pseudotyped retroviral vectors bearing the Env proteins of various FeLV strains were produced using TELCeB6 packaging cells, according to the method of Cosset et al. [25]. Briefly, TELCeB6 cells were transfected with Env expression plasmids and selected with phleomycin. Culture supernatants containing *lacZ*-pseudotyped vectors were collected, filtered through 0.45-µm membranes and stored at −80 °C until use. Viral titres were determined by counting *lacZ*-positive foci after fixation with 0.5% glutaraldehyde and staining with 1 mg/ml X-Gal and are expressed as focus-forming units (FFU) ml^−1^.

For comparative infectivity assays, virus stocks were adjusted on the basis of infectious titres before inoculation. MDCK cells in Fig. 1 were inoculated with 1.0×10^3^ FFU/well. FEA(*lacZ*) cells in Fig. 2(c) were inoculated with 1.0×10^3^ FFU/well, corresponding to an m.o.i. of 0.001. NIH3T3 cells transiently expressing THTR1 in Fig. 3 were inoculated with 1.0×10^4^ FFU/well. D17(*lacZ*) cells in Fig. 4(a were inoculated with 1.0×10^4^ FFU/well, corresponding to an m.o.i. of 0.1. For the host-range analysis shown in Table 1, virus inocula were adjusted to 1.0×10^3^ FFU/well. The FeLV strains, viral clones and controls used in this study are summarized in Table S2.

**Fig. 3.**
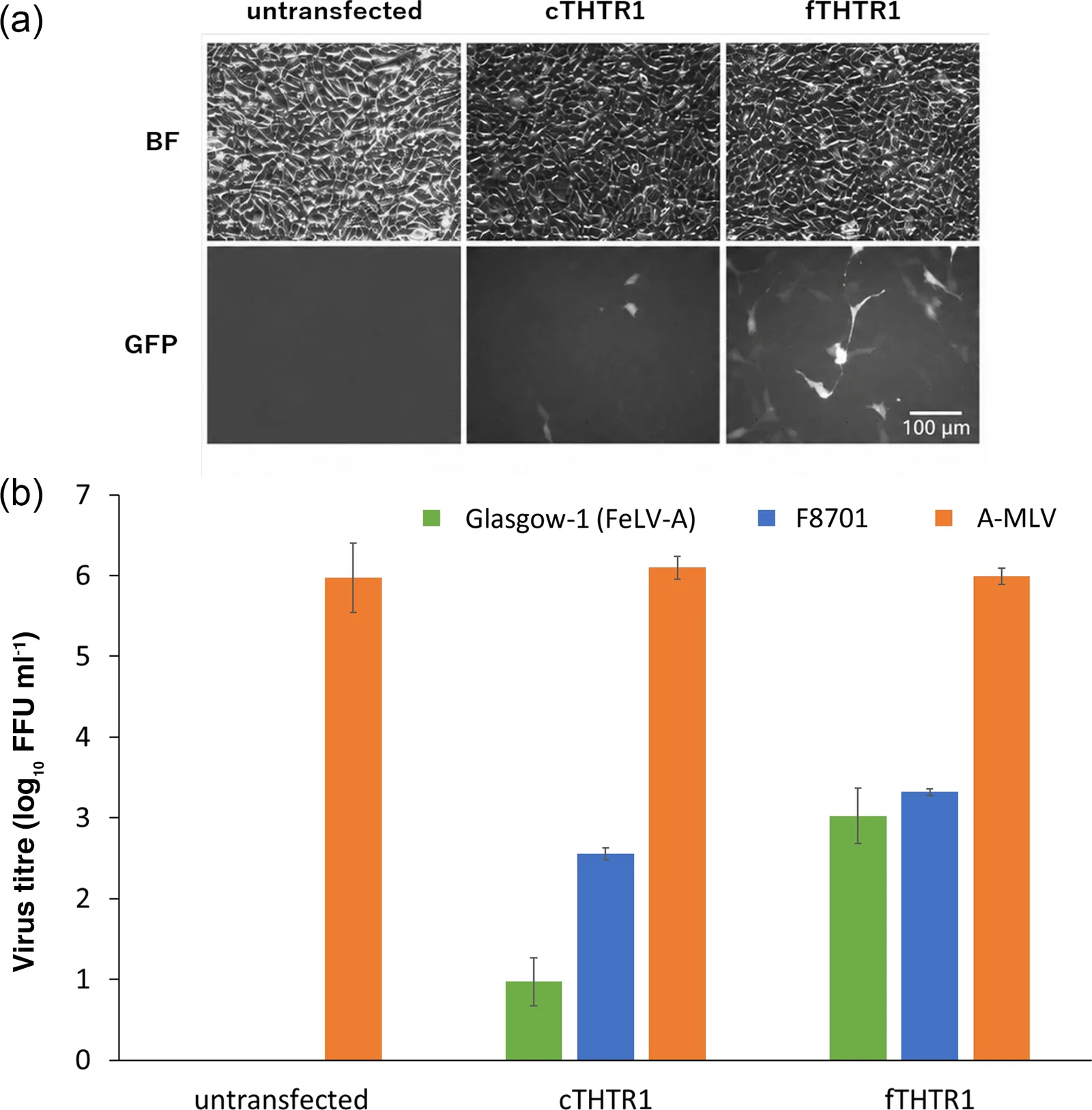
Utilization of canine THTR1 by F8701 Env. (**a**) GFP fluorescence and bright-field images of NIH3T3 cells transiently transfected with pEF-fTHTR1-2AGFP or pEF-cTHTR1-2AGFP. GFP fluorescence was used to confirm transfection of the THTR1 expression constructs, but receptor abundance was not quantitatively measured. Therefore, differences in transfection efficiency or receptor expression levels among conditions cannot be fully excluded. Scale bar, 100 µm. (**b**) Entry assay using *lacZ*-pseudotyped viruses. NIH3T3 cells transiently expressing feline THTR1 or canine THTR1 were inoculated with *lacZ*-pseudotyped viruses bearing the Env proteins of Glasgow-1 or F8701 or amphotropic MLV Env as a positive control. Viral titres were determined by counting *lacZ*-positive foci and are expressed as FFU ml^−1^. Data represent the mean±sd of technical replicate wells (*n*=4) from a representative assay.

**Fig. 4.**
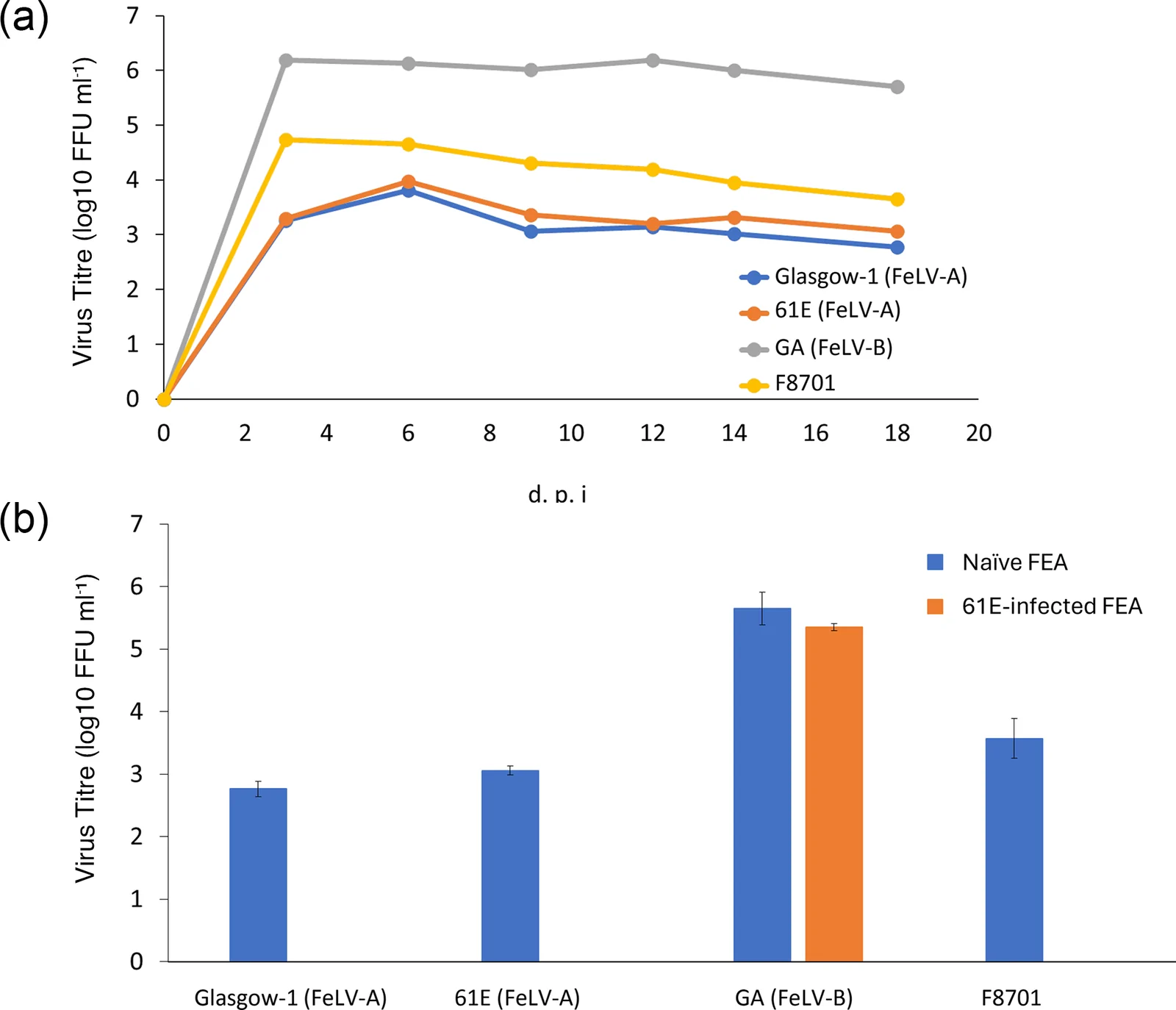
Replication kinetics of F8701 in canine D17(*lacZ*) cells and verification of subgroup identity after replication. (**a**) Growth kinetics of FeLV strains in D17(*lacZ*) cells. Cells were inoculated with FeLV-A strains Glasgow-1 and 61E, FeLV-B strain GA or F8701 at 1.0×10^4^ FFU/well, corresponding to an m.o.i. of 0.1. Culture supernatants were collected at the indicated time points, and viral titres were determined by the LacZ marker rescue assay. Data are from a single growth-kinetics experiment; each titre was determined by the LacZ marker rescue assay using technical replicate wells (*n*=4). (**b**) Interference assay of viruses recovered from D17(*lacZ*) cells. Culture supernatants collected at 18 days post-inoculation were inoculated onto naïve FEA cells or FEA cells persistently infected with FeLV-A strain 61E. Complete interference by FeLV-A-infected FEA cells indicates that the virus recovered after replication in D17(*lacZ*) cells retained the FeLV-A interference phenotype.

**Table 1.** Host cell range of FeLV strains and A-MLV in LacZ pseudotype assays Viral titres were determined by counting *lacZ*-positive foci and are expressed as FFU ml^−1^. Values represent the mean of technical replicate wells (*n*=4) from a representative LacZ pseudotype assay. ‘<5’ indicates titres below the limit of detection. Rows are grouped by host species, with alternate light grey shading used in the PDF to improve readability.

Host species	Cell line	FeLV-A Glasgow-1	F8701	A-MLV
Cat	FEA	5,500,000	5,000,000	1,500,000
Cat	CRFK	2,050,000	1,150,000	800,000
Cat	AH927	600,000	450,000	55,000
Human	TE671	65	1,000,000	300,000
Human	293T	85,000	650,000	525,000
Dog	D17	15	10,000	650,000
Dog	Cf2Th	2250	60,000	850,000
Dog	MDCK	5	35,000	140,000
Cow	MDBK	<5	8000	<5
Sheep	OKBJ	9500	950,000	8000
Horse	FHK-T cl3.1	80,000	400,000	200,000
Pig	PK15	1950	65,000	3500
Rabbit	SIRC	15	35,000	230,000
Rat	C6	<5	245	130,000
Rat	HSN	<5	<5	900,000
Mouse	MDTF	<5	<5	600,000
Mouse	NIH3T3	<5	<5	400,000
Mink	Mv1-Lu	4000	60,000	35,000
Chicken	DF-1	2950	135,000	205,000

### Focus assay using S+L− cells

The focus assay was performed to determine viral titres based on focus formation at 7 days post-inoculation, as described previously [26]. Briefly, feline sarcoma-positive leukaemia-negative S+L− QN10S cells were seeded at a density of 2×10^4^ cells/well in 24-well plates 1 day before infection. Serially diluted samples of *lacZ*-pseudotyped viruses in 500 µl of DMEM growth medium were inoculated onto cells in the presence of 8 µg ml^−1^ polybrene. At 7 days post-inoculation, foci became prominent, and viral titres were determined as FFU ml^−1^.

### LacZ marker rescue assay

The LacZ marker rescue assay was performed as previously described [19]. Briefly, feline FEA cells and canine D17 cells were transduced with the *nlslacZ* gene to generate FEA(*lacZ*) and D17(*lacZ*) indicator cells. These cells constitutively express *lacZ* mRNA containing a murine leukaemia virus (MLV) packaging signal. Because FeLV core proteins are derived from a gammaretrovirus, similar to MLV, the *lacZ* mRNA can be co-packaged into FeLV particles and ‘rescued’ as infectious vector RNA. To monitor FeLV replication, culture supernatants were collected at several time points, passed through a 0.45-µm filter and immediately inoculated onto naïve FEA cells. Two days after inoculation, cells were stained with X-Gal, and *lacZ*-positive foci were counted. To exclude the possibility of contamination or non-specific rescue, supernatants from FeLV-infected D17(*lacZ*) cells were inoculated into both naïve FEA cells and FEA cells previously infected with FeLV-A strain 61E, followed by X-Gal staining and foci counting 3 days later.

### Analysis of canine THTR1 as an entry receptor

To investigate receptor usage by F8701, NIH3T3 cells, which are inherently non-susceptible to FeLV-A infection because they lack a functional FeLV-A receptor, were seeded at a density of 5.0×10^5^ cells/well in 6-well plates. The following day, cells were transfected with pEF-cTHTR1-2AGFP or pEF-fTHTR1-2AGFP. Eight hours after transfection, transfected and untransfected cells were inoculated with *lacZ*-pseudotyped viruses bearing the Env proteins of Glasgow-1 or F8701, or amphotropic murine leukaemia virus (A-MLV) Env as a positive control. Three days after infection, cells were stained with X-Gal, and *lacZ*-positive foci were counted. Expression from the pEF-THTR1-2AGFP vectors was assessed by observing GFP fluorescence in transfected NIH3T3 cells using an IX71-23XL fluorescence microscope (Olympus, Tokyo, Japan) 2 days after transfection. GFP fluorescence was used to confirm transfection of the THTR1 expression constructs, but receptor abundance was not quantitatively measured.

### Sequence alignment and phylogenetic analysis

Amino acid sequences of FeLV Env proteins were retrieved from the GenBank database for the following strains: FeLV-A Glasgow-1 (KP728112 [27]), FeLV-A 61E (M18247 [28]), FeLV-B Gardner-Arnstein (X00188 [29]), FeLV-C Sarma (M14331 [30]), FeLV-A/C FY981 (FJ436991 [10]), FeLV-D ON-T (AB673426 [4]) and FeLV-E TG35-2 (AB635781 [7]). The Env nt sequence of the molecular clone derived from FeLV strain F8701 has been deposited in DDBJ under accession number LC939534. The DDBJ entry uses the entry identifier F87A1_env; for clarity and consistency, the molecular clone is designated pF8701 in the present manuscript.

Env amino acid sequences were aligned using Clustal Omega [31]. Residues in VRA were numbered according to the numbering system used by Stewart *et al*. [14], and residues at positions 83, 91 and 105 were highlighted in the alignment.

Phylogenetic analysis was performed using IQ-TREE3 v3.0.1 [32] based on the maximum-likelihood method. The best-fit amino acid substitution model was determined as FLU+G4 based on the Bayesian Information Criterion. Branch support was assessed using 1,000 ultrafast bootstrap replicates. The tree was visualized using FigTree v1.4.4 (http://tree.bio.ed.ac.uk/software/figtree/).

## Results

### Parental FeLV strain F8701 infects canine MDCK cells

We first examined whether the parental F8701 isolate could infect canine cells. MDCK cells were inoculated with FeLV-A strains Glasgow-1 and 61E, FeLV-B strain GA or virus-containing supernatant from FEA cells infected with the parental FeLV strain F8701. Virus inocula were adjusted to 1.0×10^3^ FFU/well on the basis of infectious titres, and infection was monitored by real-time PCR targeting the U3 region of the FeLV LTR. Because the assay detects FeLV LTR DNA, the results are described as FeLV LTR DNA copy numbers rather than as proviral DNA copy numbers.

At 9 days post-inoculation, F8701 produced a marked increase in FeLV LTR DNA copy numbers in MDCK cells (Fig. 1a). Endpoint PCR amplification of the env region also detected an approximately 2-kbp product in DNA extracted from F8701-infected MDCK cells at this time point (Fig. 1b). In contrast, the reference FeLV-A strains Glasgow-1 and 61E produced only low levels of detectable FeLV DNA under the same conditions. These results indicate that the parental F8701 isolate can infect canine MDCK cells more efficiently than the reference FeLV-A strains tested.

### Construction and characterization of an infectious molecular clone of FeLV strain F8701

To characterize the biological properties of FeLV strain F8701 under genetically defined experimental conditions, we constructed an infectious molecular clone derived from the F8701 isolate. Two overlapping genomic fragments covering the entire FeLV genome were amplified from genomic DNA of F8701-infected FEA cells and cloned into pSP73 to generate pF8701 (Fig. 2a). To avoid unnecessary nomenclatural complexity, the molecular clone is designated pF8701, and the virus rescued from this clone is referred to as F8701 throughout this manuscript unless otherwise specified.

The clone-derived F8701 virus replicated efficiently in feline FEA cells. In the S+L− focus assay, F8701 reached titres exceeding 10^5^ FFU ml^−1^ at 14 days post-transfection, comparable to the reference FeLV-A strains Glasgow-1 and 61E (Fig. 2b). To further compare replication kinetics in feline cells, FEA(*lacZ*) cells were inoculated with Glasgow-1, 61E and F8701 at 1.0×10^3^ FFU/well, corresponding to an m.o.i. of 0.001, and viral titres in culture supernatants were monitored by the LacZ marker rescue assay for 18 days. F8701 reached a plateau of ~10^7.0^ FFU ml^−1^ at 9 days post-inoculation and showed replication kinetics similar to those of the reference FeLV-A strains (Fig. 2c). These results indicate that clone-derived F8701 is replication-competent in feline cells and grows comparably to established FeLV-A strains.

### Analysis of amino acid sequences in the Env VRA and phylogenetic relationship of F8701

We next analysed the Env amino acid sequence of F8701, focusing on VRA, which is known to influence FeLV-A receptor usage and host range. The Env sequence of F8701 showed high overall similarity to those of reference FeLV-A strains but differed at several positions within VRA (Fig. 5a). In particular, F8701 carried aspartic acid residues at positions 83 and 91. This 83D/91D di-aspartic acid residue combination has previously been associated with enhanced utilization of mammalian THTR1 orthologues and expanded infectivity in non-feline cells.

**Fig. 5.**
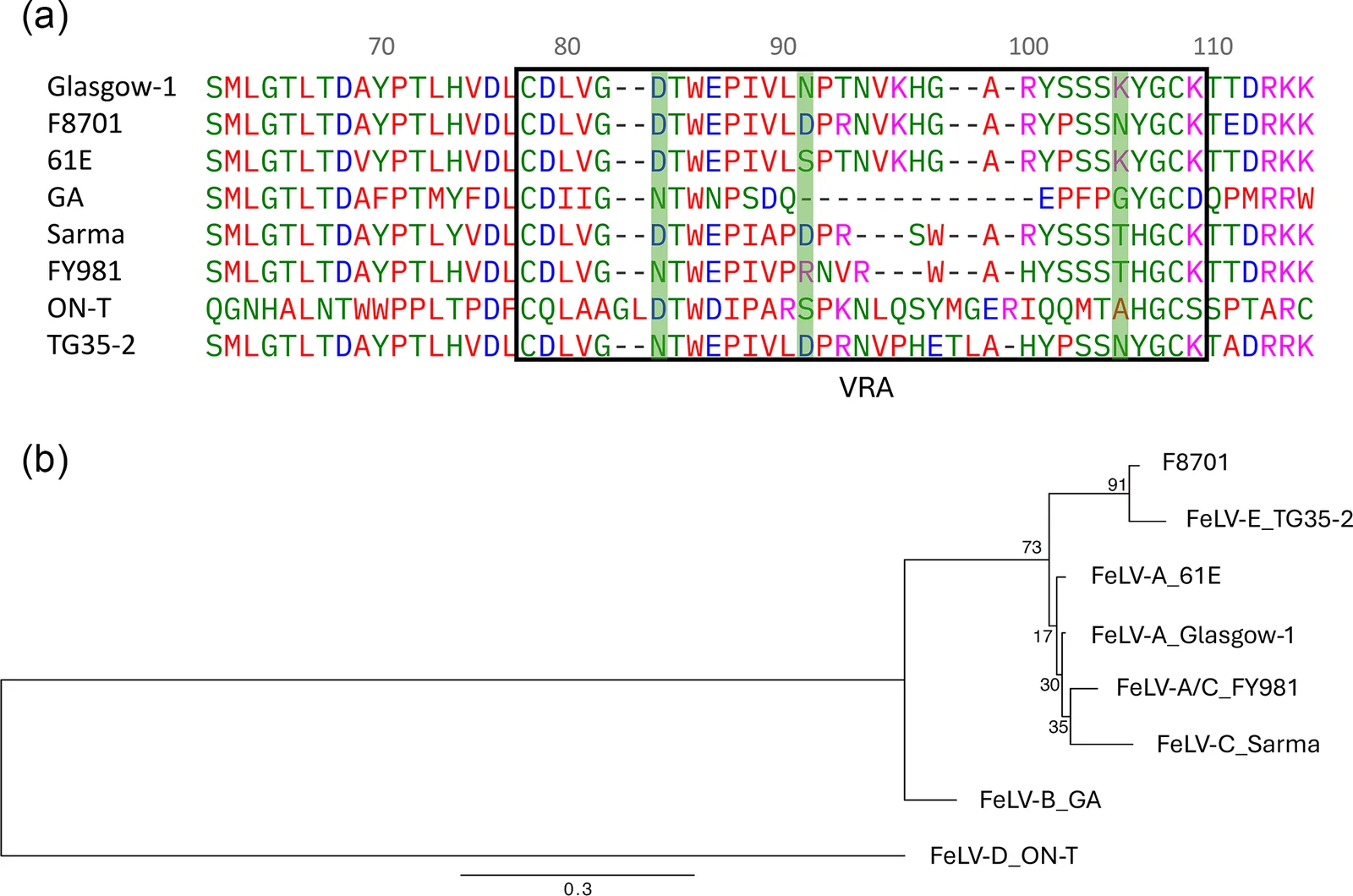
Amino acid alignment and phylogenetic analysis of FeLV Env proteins. (**a**) Partial Env amino acid alignment including variable region A (VRA) for FeLV-A strains Glasgow-1, 61E and F8701, FeLV-B strain GA, FeLV-C strain Sarma, FeLV-A/C strain FY981, FeLV-D strain ON-T and FeLV-E strain TG35-2. Residue numbering is based on Stewart *et al*. [14]. Positions 83, 91 and 105 are highlighted. F8701 carries the previously described 83D/91D di-aspartic acid residue combination together with an additional 105 N substitution. The boxed region indicates VRA. (**b**) Maximum-likelihood phylogenetic tree of FeLV Env proteins. Numbers at internal nodes indicate ultrafast bootstrap support values (%) based on 1,000 replicates. The scale bar represents 0.3 amino acid substitutions per site.

In addition to the 83D/91D residue combination, F8701 carried an asparagine residue at position 105, whereas several reference FeLV-A strains possess lysine at this position. Thus, the VRA of F8701 is characterized by the combination of 83D, 91D and 105 N. Because the functional contribution of residue 105 was not examined by site-directed mutagenesis in the present study, the role of 105 N in the expanded cellular tropism of F8701 remains to be determined.

To place F8701 in the context of representative FeLV Env sequences, we also constructed a maximum-likelihood phylogenetic tree based on Env amino acid sequences (Fig. 5b). In this analysis, F8701 was positioned within the group containing FeLV-A, FeLV-C, FeLV-E and FeLV-A/C Env sequences, whereas FeLV-B GA and FeLV-D ON-T were more distant. F8701 formed a supported branch with FeLV-E strain TG35-2 in this tree. These phylogenetic data provide sequence context for F8701 Env, while the interference assays described below were used to determine its functional interference subgroup.

### Broad cellular host range of F8701

To determine the extent of the cellular host range of F8701, we performed infectivity assays using *lacZ*-pseudotyped retroviral vectors bearing the Env proteins of F8701 or reference FeLV strains. A panel of mammalian and avian cell lines was used, including feline, canine, human, bovine, ovine, equine, porcine, rabbit, rat, mouse, mink and avian cells (Table 1). Values in Table 1 represent the mean of technical replicate wells from a representative *lacZ* pseudotype assay.

F8701 Env conferred a broad cellular host range in this pseudotype assay. Compared with Glasgow-1 Env, F8701 Env yielded markedly higher titres in several non-feline mammalian cell lines. Notably, *lacZ*(F8701) showed ~15,000-fold higher infectivity in human TE671 cells, ~100-fold higher infectivity in ovine OKBJ cells and ~7,000-fold higher infectivity in canine MDCK cells than *lacZ*(Glasgow-1). These findings indicate that F8701 Env permits efficient entry into multiple non-feline mammalian cell types, rather than showing an expansion limited to canine cells.

### Utilization of canine THTR1 by F8701

We then examined whether the expanded infectivity of F8701 in canine cells was associated with utilization of canine THTR1, the orthologue of the canonical FeLV-A receptor. NIH3T3 cells, which are inherently resistant to FeLV-A infection because they lack a functional FeLV-A receptor, were transiently transfected with expression plasmids encoding canine THTR1 or feline THTR1. These constructs also express GFP through a 2A sequence, allowing transfected cells to be visualized by fluorescence microscopy (Fig. 3a). GFP fluorescence was used to confirm transfection of the THTR1 expression constructs, but receptor abundance was not quantitatively measured.

After transfection, cells were inoculated with *lacZ*-pseudotyped viruses bearing the Env proteins of Glasgow-1 or F8701 or amphotropic MLV Env as a positive control. In cells expressing canine THTR1, *lacZ*(F8701) produced markedly higher titres than the pseudotype bearing Glasgow-1 Env (Fig. 3b). In contrast, both F8701 and Glasgow-1 Envs mediated infection of cells expressing feline THTR1. These results support the interpretation that F8701 can utilize canine THTR1 as an entry receptor more efficiently than the reference FeLV-A strain tested. However, because receptor expression was assessed by GFP fluorescence rather than by quantitative measurement of receptor abundance, possible effects of differences in transfection efficiency or receptor expression level cannot be fully excluded.

### F8701 belongs to interference subgroup A

To determine the interference subgroup of F8701, we performed receptor interference assays using feline FEA cells. Uninfected FEA cells and FEA cells persistently infected with FeLV-A strain Glasgow-1 or F8701, FeLV-B strain GA, or FeLV-C strain Sarma were inoculated with lacZ-pseudotyped viruses bearing the Env proteins of F8701, Glasgow-1, GA or Sarma (Fig. 6). A-MLV was included as a positive control for infection of the indicator cells. Both *lacZ*(F8701) and *lacZ*(Glasgow-1) efficiently infected FEA cells and FEA cells persistently infected with FeLV-B or FeLV-C. In contrast, infection by both pseudotypes was blocked in FEA cells persistently infected with FeLV-A strain Glasgow-1 or F8701. This interference pattern indicates that F8701 uses the FeLV-A entry pathway and belongs to interference subgroup A, despite its broadened cellular host range.

**Fig. 6.**
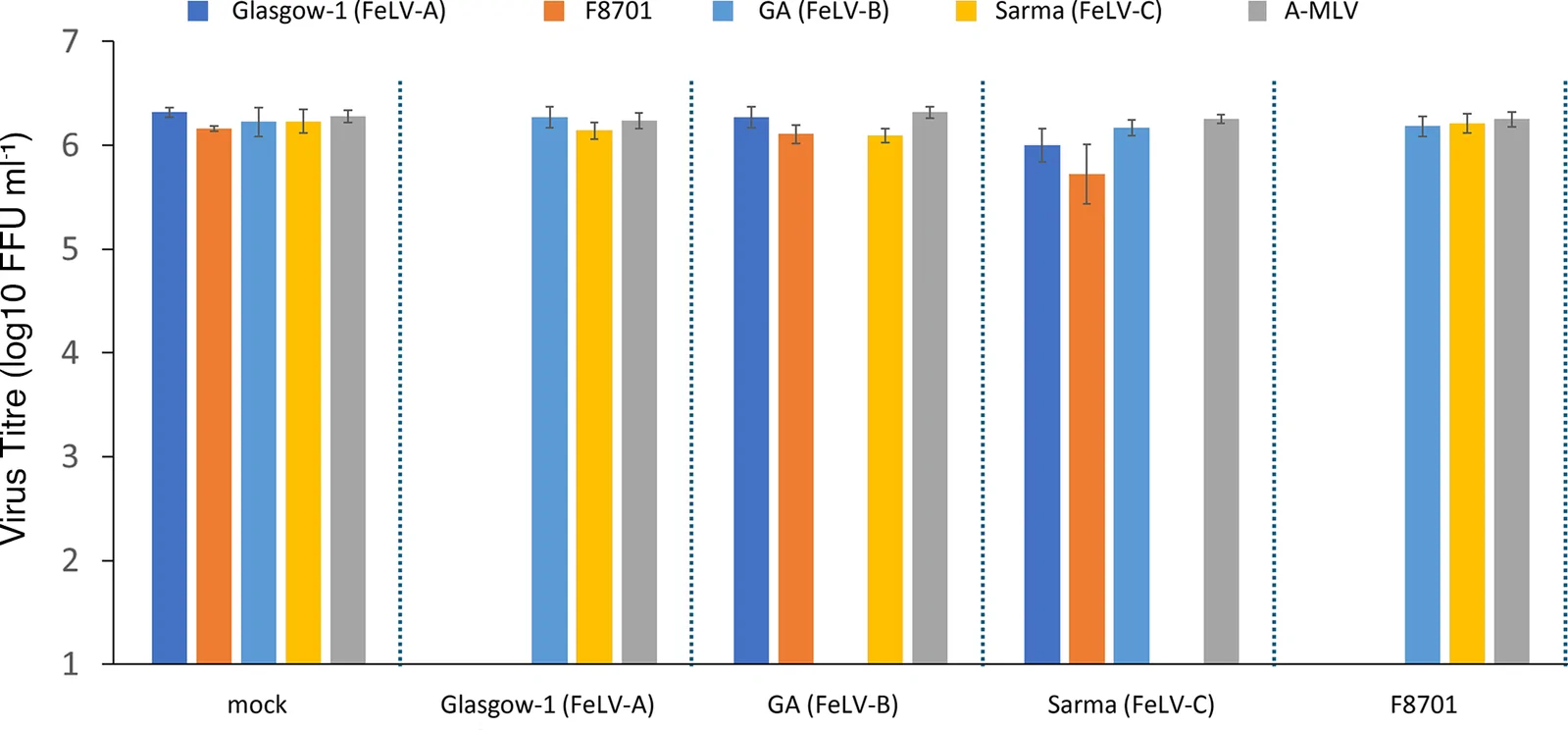
Interference analysis of F8701 Env. Uninfected FEA cells (mock) and FEA cells persistently infected with FeLV-A strain Glasgow-1 or F8701, FeLV-B strain GA, or FeLV-C strain Sarma were inoculated with lacZ-pseudotyped viruses bearing the Env proteins of Glasgow-1, F8701, GA or Sarma. A-MLV was included as a positive control. Viral titres were determined by counting lacZ-positive foci and are expressed as FFU ml⁻¹. Infection by both Glasgow-1 and F8701 pseudotypes was blocked in FEA cells persistently infected with FeLV-A strain Glasgow-1 or F8701, but not in FeLV-B- or FeLV-C-infected FEA cells, indicating that F8701 belongs to interference subgroup A. Dotted lines separate groups of bars corresponding to the different cell conditions. Data represent the mean±sd of technical replicate wells (n=4) from a representative assay.

### Replication kinetics of F8701 in canine cells and verification of strain purity

To evaluate whether F8701 can replicate in canine cells, we examined its growth kinetics in D17(*lacZ*) cells. Cells were inoculated with FeLV-A strains Glasgow-1 and 61E, FeLV-B strain GA or F8701 at 1.0×10^4^ FFU/well, corresponding to an m.o.i. of 0.1, and viral titres in culture supernatants were monitored for 18 days by the LacZ marker rescue assay (Fig. 4a).

F8701 replicated efficiently in D17(*lacZ*) cells and reached a high-titre plateau as early as 3 days post-inoculation. In contrast, Glasgow-1 and 61E showed slower replication kinetics and reached lower peak titres under the same conditions. These results indicate that F8701 can undergo productive replication in cultured canine D17 cells more efficiently than the reference FeLV-A strains tested.

To rule out the possibility that the observed replication in canine cells resulted from cross-contamination with FeLV-B or another FeLV subgroup, we collected culture supernatants from infected D17(*lacZ*) cells at 18 days post-inoculation and subjected them to an interference assay using FEA cells persistently infected with FeLV-A strain 61E (Fig. 4b). Virus recovered from F8701-infected D17(*lacZ*) cells was completely interfered with by FeLV-A 61E-infected FEA cells. This result confirms that the replicated virus retained the FeLV-A interference phenotype and that detectable replication in D17(*lacZ*) cells was not attributable to contamination with another FeLV subgroup.

## Discussion

In this study, we characterized a naturally occurring FeLV-A isolate, F8701, that exhibits broad mammalian cellular tropism compared with prototypical FeLV-A strains. Initial analysis using the parental F8701 isolate showed efficient infection of canine MDCK cells. We then constructed an infectious molecular clone of F8701 and showed that the clone-derived virus replicated efficiently in feline cells, infected canine cells more efficiently than reference FeLV-A strains and retained the interference phenotype of subgroup A. These findings indicate that substantial expansion of cellular host range can occur within a naturally occurring FeLV-A background, without conversion to another interference subgroup.

The expanded cellular tropism of F8701 is consistent with previous work showing that the FeLV-A Env VRA is a key determinant of receptor usage and host range. In particular, a di-aspartic acid residue combination at positions 83 and 91 in VRA has been associated with enhanced utilization of mammalian THTR1 orthologues and infection of non-feline cells [14]. F8701 carries this 83D/91D residue combination, providing a plausible explanation for its increased infectivity in several non-feline mammalian cell lines. The present results therefore support the view that limited natural variation in the FeLV-A Env protein can markedly alter cellular tropism.

F8701 also carries an additional K105N substitution in VRA. Because residue 105 differs between F8701 and several reference FeLV-A strains, it may be relevant to the distinctive biological properties of F8701. However, the functional contribution of this residue was not directly tested in the present study. In particular, we did not perform site-directed mutagenesis to revert 105 N in the F8701 backbone or to introduce 105 N into a reference FeLV-A Env. Therefore, the role of 105 N remains unresolved, and the present data should not be interpreted as demonstrating a causal contribution of this residue to the broad cellular tropism of F8701. Further mutational analyses will be required to determine whether 105 N modulates receptor interaction, Env conformation or infectivity in particular host cell types.

The phylogenetic analysis of Env proteins provides additional context for interpreting F8701. F8701 was positioned among FeLV-A-related Env sequences and was separated from FeLV-B GA and FeLV-D ON-T, supporting the view that F8701 is not a FeLV-B- or FeLV-D-like Env variant. At the same time, the Env tree alone does not define the interference subgroup of a virus. Therefore, the phylogenetic analysis should be interpreted together with the receptor interference assays. In this framework, F8701 is best interpreted as a naturally occurring FeLV-A variant that retains subgroup A interference properties while exhibiting expanded mammalian cellular tropism.

A notable feature of F8701 is that its expanded infectivity was not restricted to canine cells. In pseudotype assays, F8701 Env mediated markedly higher infectivity than Glasgow-1 Env in multiple non-feline mammalian cell lines, including human, ovine, porcine and canine cells. This observation suggests that F8701 is not simply a canine-adapted variant, but rather a broad-tropic FeLV-A variant at the level of cellular entry. At the same time, the receptor determinants responsible for infection of each non-feline cell type remain to be defined. In the present study, we focused on canine THTR1 because F8701 infected canine cells efficiently and because canine cells provide a clear model for testing expansion beyond the feline host range. Analysis of additional THTR1 orthologues, including human THTR1, will be important in future studies.

The receptor expression experiment supports the interpretation that F8701 can utilize canine THTR1 as an entry receptor more efficiently than the reference FeLV-A strain under the conditions tested. Nevertheless, this conclusion has limitations. Expression of the THTR1 constructs was assessed by GFP fluorescence from the 2A-linked expression cassette, rather than by quantitative measurement of receptor protein levels. Therefore, possible differences in transfection efficiency or receptor abundance between feline and canine THTR1 constructs cannot be fully excluded. For this reason, we interpret the receptor expression data as supporting the ability of F8701 to use canine THTR1, while recognizing that more quantitative approaches, such as flow cytometry or immunoblotting, would further strengthen this conclusion.

The ability of F8701 to replicate in cultured canine D17 cells is also important for interpreting the biological significance of its expanded entry phenotype. These data indicate that at least some canine cells can support post-entry steps of F8701 replication *in vitro*. However, replication in cultured canine cells does not necessarily imply that F8701 can establish persistent infection, cause disease or undergo transmission in dogs *in vivo*. Host susceptibility at the organismal level depends not only on receptor compatibility and replication in individual cell lines but also on tissue distribution of susceptible cells, innate and adaptive immune responses, viral spread within the host and other post-entry restrictions.

Post-entry restriction factors may therefore remain relevant to the effective host range of F8701, even though replication was observed in cultured canine cells. FeLV encodes a glycosylated form of Gag, Glyco-Gag, which is conserved among gammaretroviruses and has been implicated in counteracting host restriction mechanisms [33–35]. In FeLV, Glyco-Gag has also been reported to contribute to resistance against SERINC proteins, although this effect appears to depend on the viral Env context [36]. Whether FeLV Glyco-Gag can efficiently counteract canine SERINC proteins or canine APOBEC3 proteins remains unknown. Thus, while our findings show that F8701 can replicate in cultured canine cells, they do not exclude the possibility that host restriction factors may limit infection, persistence or spread *in vivo*.

Taken together, the present study demonstrates that a naturally occurring FeLV-A isolate can exhibit broad mammalian cellular tropism while retaining subgroup A interference properties. The data support a model in which natural Env variation, particularly within VRA, can expand receptor compatibility and cellular entry range. At the same time, the findings emphasize the distinction between cellular tropism and host-level susceptibility. Receptor usage and entry efficiency are important determinants of host range, but they are not sufficient by themselves to predict cross-species infection or transmission *in vivo*. F8701 therefore provides a useful natural model for dissecting how Env variation, receptor compatibility and post-entry host factors together shape the evolutionary potential of gammaretroviruses.

## Supplementary material

10.1099/jgv.0.002316 Supplementary Material 1.

## Abbreviations

A-MLV: amphotropic murine leukaemia virus
DMEM: Dulbecco’s modified Eagle’s medium
FeLV: feline leukaemia virus
FeLV-A: feline leukaemia virus subgroup A
FFU: focus-forming units
LTR: long terminal repeat
MDCK: Madin–Darby canine kidney
MLV: murine leukaemia virus
THTR1: thiamine transporter 1
VRA: variable region A
